# Novel device for dividing core needle biopsy specimens to provide paired mirror image-like tissues for genetic and pathological tests

**DOI:** 10.1038/s41598-023-33776-x

**Published:** 2023-04-24

**Authors:** Yuichi Nakamura, Keisuke Tsuji, Takumi Shiraishi, Satoshi Sako, Ryota Ogura, Hideto Taga, Yuta Inoue, Munehiro Ohashi, Saya Ueda, Takeshi Yamada, Takashi Ueda, Atsuko Fujihara, Fumiya Hongo, Osamu Ukimura

**Affiliations:** Department of Urology, Graduate School of Medical Science, Kyoto Prefectural University of Medicine, Kawaramachi-Hirokoji, Kamigyo-Ku, Kyoto, 602-8566 Japan

**Keywords:** Prostate cancer, Histology

## Abstract

In a world that seeks precision medicine, genetic testing is gaining importance in clinical decision making. We previously reported the utility of a novel tool for longitudinally dividing core needle biopsy (CNB) tissues into two filamentous tissues that can provide paired mirror image-like tissues (mirror-tissues) that spatially match each other. In this study, we investigated its application in gene panel testing in patients who underwent prostate CNB. Four hundred and forty-three biopsy cores were obtained from 40 patients. Of them, 361 biopsy cores (81.5%) were judged by a physician to be appropriate for dividing into two pieces using the new device, of which a histopathological diagnosis was successfully reached in 358 biopsy cores (99.2%). Among them, the quality and quantity of nucleic acid in 16 appropriately divided cores were assessed and found to be sufficient for gene panel testing, and histopathological diagnosis was successfully obtained from the remaining divided cores. The novel device for longitudinally-dividing CNB tissue provided mirror image-like paired-tissues for gene panel and pathology testing. The device might be a promising tool for obtaining genetic and molecular biological information, in addition to histopathological diagnosis, helping to advance personalized medicine.

## Introduction

Prostate biopsy is still the mandatory procedure for the diagnosis or exclusion of prostate cancer (PCa). Even with an increasing use of valid, sensitive, and reliable diagnostic tools such as imaging techniques and urine biomarkers, tissue-based analysis and decision making are indispensable for both the management and prognosis of PCa^1^. According to the current trends, the management strategies are dependent on the clinical and histopathological presentation that comprises of clinical staging, serum prostate specific antigen (PSA) level and density, digital rectal examination, and the Gleason score of biopsy specimens. Patients with PCa are offered various management regimens based on their risk group. These treatment options could be appropriate in a certain number of patients. However, it is important to acknowledge that these treatment strategies may not be always most suitable for each individual patient, where some potentially low-risk patients classified in high-risk group may receive unnecessary management (overtreatment) and some potentially high-risk patients in low-risk group may delayed treatment onset^2^.

The oncogenesis of PCa is associated with many factors, such as innate germline susceptibility and acquired somatic gene alterations, in addition to environmental factors. Furthermore, progression to metastatic castration-resistant PCa (mCRPC) is related to impaired regulation of additional genes manifesting in growth control and genetic stability^3^. The novel methods such as genomics, epigenomics, transcriptomics and proteomics using different types of samples (tissue biopsies, plasma and urine) could aid in identifying these factors, leading to the identification of specific drugs that can effectively target the molecular pathways altered due to cancer as well as the determination of prognostic tools and predictive biomarkers. Such advances can support better treatment selection and patient identification for specific therapy and offer a tailor-made regimen in PCa management. Thus, obtaining appropriate biopsy samples, along with advances in genomic sequencing, have provided novel insights into the molecular overview of CRPC, selection of actionable targets, and emergence of resistance mechanisms^4^.

Recent research has greatly increased our knowledge of diagnosis, prognosis, and treatment of PCa at a molecular level, and treatment of a poly ADP ribose polymerase (PARP) inhibitor or pembrolizumab could be applied in PCa patients with homologous recombination repair gene mutations or mismatch repair defects/microsatellite instability. As a matter of fact, genetic testing to recognize advanced and/or high-risk PCa patients harboring these genetic alterations are recommended by current international guidelines^5^. Thus, in future, the treatment of PCa will be significantly relying on complete understanding of the molecular mechanisms that directs the therapeutic efficacy. Precision medicine where unique treatment methodologies in every patient could be identified by seeing every gene or pathway that is modified in a patient may lead to the improvement of patient outcomes^6^.

In this context, we recently developed a novel device for longitudinally-dividing CNB specimens into two filamentous tissues, which would provide a pair of mirror image-like tissue samples (mirror-tissues) that spatially match each other, with one being used for molecular-biological analysis including genetic testing, and the other for pathological diagnosis, to identify the location of disease that spatially matches that in the other mirror-tissue^7^. In this study, we investigated its application in prostate CNB in patients with suspected PCa and found that the new device has the potential to be a promising tool for obtaining appropriate tissue for genetic and molecular biological analyses, leading to advance personalized medicine.

## Results

### Characteristics of patients and samples

**Figure 1:**
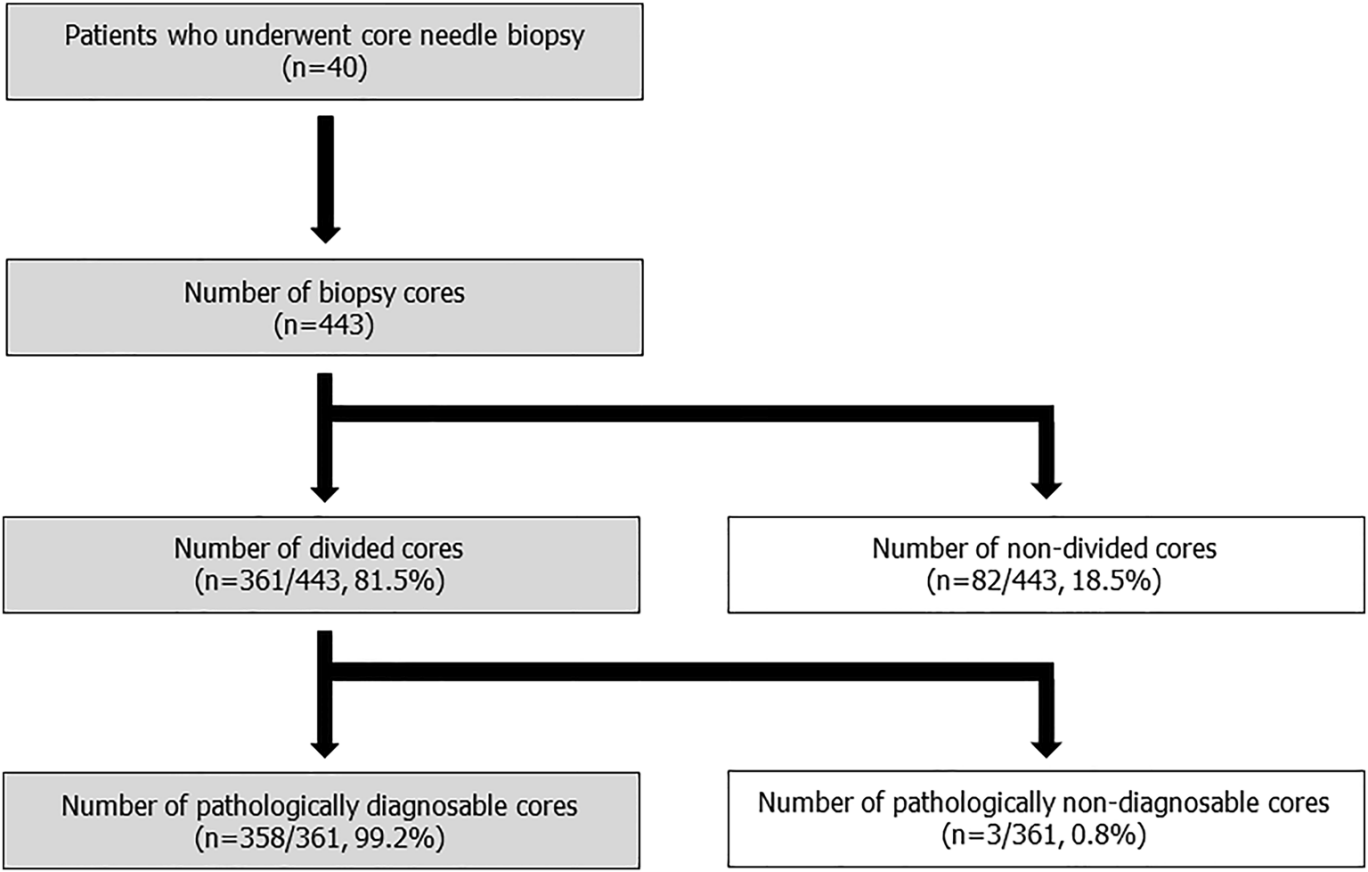
Patient and sample selection. Of a total of 443 biopsy cores from 40 patients, 361 biopsy cores (81.5%) were divided into two pieces using the new device. Of these, histopathological diagnosis was successfully made in 358 biopsy cores (99.2%).

Four hundred and forty-three biopsy cores were obtained from 40 patients. MRI/Ultrasound fusion targeted biopsy and systematic biopsy were conducted in 31 (77.5%) and 9 (22.5%) patients, respectively. Of the 443 biopsy cores, 361 cores (81.5%) were judged by a physician to be appropriate for dividing into two pieces using the new device. Although three biopsy cores from two patients (0.8%) could not be histopathologically diagnosed due to the small volume of tissue, a histopathological diagnosis was successfully reached in the remaining 358 biopsy cores (99.2%) (Fig. 1). Patient background characteristics are described in Table 1. Median age of the patients was 73 years (range 49–91) and median PSA levels was 7.84 ng/mL (range 0.49–4504). The median number of total biopsy cores and divided cores per patient were 12 (range 4–15) and 10 (range 2–14), respectively. Of the 40 patients, 28 (70.0%) cases were diagnosed with PCa, with a Gleason score of 7 in 10 (35.7%) cases, 8 in nine (32.1%) cases, 9 in eight (28.6%) cases, and 10 in one (3.6%) case.

**Table 1:** Patients’ characteristics.

Factor	Group	Overall (n = 40)
Age, years (median, [range])		73 [49, 91]
PSA, ng/mL (median [range])		7.84 [0.49, 4504.00]
Median prostate volume (mL, [range])		38.5 [13.0, 210.0]
PI-RADS score, n (%)	3	6 (17.1)
	4	16 (45.7)
	5	13 (37.1)
Biopsy method, n (%)	MRI/US fusion	31 (77.5)
	Systematic	9 (22.5)
Total number of biopsy cores		443
Median number of biopsy cores/patient (n, [range])		12 [4, 15]
Total number of divided biopsy cores, n (%)		361/443 (81.5)
Median number of divided cores/patient (n, [range])		10 [2, 14]
Diagnosed with cancer, n (%)		28 (70.0)
Median core length (mm, [range])		11.0 [1.0, 22.0]
Median cancer core length (mm, [range])		6.0 [0.2, 20.0]
ISUP Grade Group, n (%)	2	7/28 (25.0)
	3	3/28 (10.7)
	4	9/28 (32.1)
	5	9/28 (32.1)

PSA = prostate specific antigen; PI-RADS = prostate imaging-reporting and data system; MRI = magnetic resonance imaging; US = ultrasound; ISUP = International Society of Urological Pathology.

### Gene panel testing

To investigate whether the divided samples obtained using the new device were suitable for gene panel testing, we performed two kinds of gene panel tests obtained from two different companies in 16 appropriately-divided samples. The amount of DNA obtained from the divided samples varied depending on the sample, although the quality and quantity of all 16 divided specimens were sufficient for gene panel testing (Tables 2 and 3). Furthermore, a histopathological diagnosis was successfully obtained using the rest of the divided cores in all 16 specimens and structural artefacts were not introduced microscopically by the core splitting (Supplemental Fig. 1).

**Table 2:** Quality assessment of divided samples for gene panel testing (Oncomine Comprehensive Assay v3).

Sample ID	Fluorometric quantification		qPCR quantification		Deviation value	Quality assessment
	Concentration (ng/µL)	Total amount (ng)	Concentration (ng/µL)	Total amount (ng)		
TA_001	10.7	438.7	8	328	0.75	Pass
TA_002	0.5	16.7	N/A	N/A	N/A	Pass
TA_003	33	1188	22.6	813.6	0.68	Pass
TA_004	46.2	1663.2	32.5	1170	0.7	Pass
TA_005	36.4	1237.6	11.8	401.2	0.32	Pass
TA_006	72.4	2606.4	42.5	1530	0.59	Pass

qPCR = quantitative polymerase chain reaction.

**Table 3:** Quality assessment of divided samples for gene panel testing (TruSight Oncology 500).

Sample ID	Liquid volume (µL)	Qubit concentration (ng/µL)	Qubit DNA quantity (ng)	DIN	Quality assessment
TS_001	48	23.2	1113.6	8.6	Pass
TS_002	48	12.8	614.4	8.4	Pass
TS_003	48	4.2	201.6	8.3	Pass
TS_004	48	38.2	1833.6	8.8	Pass
TS_005	48	3.2	153.6	7.2*2	Pass
TS_006	48	15.6	748.8	8.5	Pass
TS_007	48	23.4	1123.2	8.6	Pass
TS_008	48	10.4	499.2	8.6	Pass
TS_009	48	19	912	8.7	Pass
TS_010	48	11.2	537.6	8.6	Pass

DIN = DNA integrity number.

### Trimming of tissues to increase tumor cell content ratio

**Figure 2:**
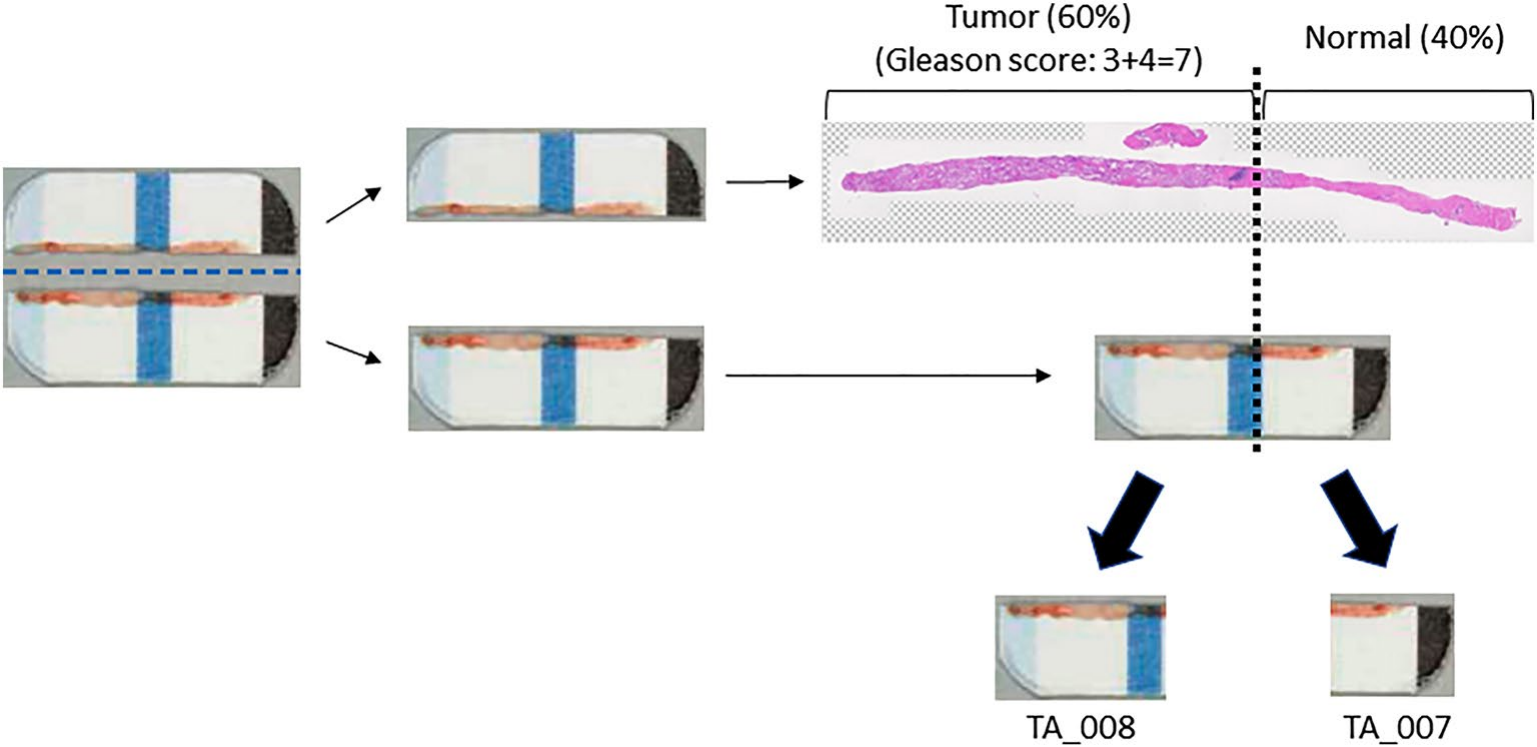
Representative images of trimming of a sample. To increase tumor cell content ratio, the divided sample is trimmed with reference to histopathological information.

Tumor cell content ratio is important for gene panel testing, and a minimum tumor content rate of 20% is required to obtain reliable results. Therefore, we examined whether tumor cell content ratio can be increased by trimming a fresh-frozen divided sample based on histopathological information from another spatially matched sample. Figure 2 demonstrates trimming of the sample. In this study, we further split a divided frozen sample into two parts with reference to pathological information, as shown in Fig. 2. As described in Table 4, the quality and quantity of the trimmed samples were adequate for performing gene panel testing.

**Table 4:** Quality assessment of divided samples with trimming for gene panel testing.

Sample ID	Fluorometric quantification		qPCR quantification		Deviation value	Quality assessment
	Concentration (ng/µL)	Total amount (ng)	Concentration (ng/µL)	Total amount (ng)		
TA_007	4	126	2.6	81.9	0.65	Pass
TA_008	26.2	655	21.2	530	0.81	Pass

qPCR = quantitative polymerase chain reaction.

## Discussion

This study revealed that our novel device for longitudinally dividing CNB specimens is useful for providing fresh frozen tissue that spatially corresponds to histopathological findings in patients who undergo prostate CNB. Furthermore, we clearly showed that the new device provided high quality samples of adequate quantity to perform gene panel testing. The results of this study suggest the possibility that the new device has several advantages in terms of providing paired mirror-tissues from a single core biopsy sample that can be used for molecular biological analysis, such as performance of gene expression analysis, proteomics and transcriptomics, as well as gene panel testing.

The tissues employed in clinical testing are predominantly formalin-fixed or paraffin-embedded (FFPE). The extra edge in using FFPE tissue are handling convenience, long-term but cheap storage option, suitability for immunohistochemical analyses, and cost-effectiveness relative to its large-scale application^8,9^. The wide range of analyses of nucleic acids (NA) derived from FFPE tissue is on a rise recently and several molecular analyses, including gene panel testing, are usually performed by using NAs that are extracted from FFPE samples. However, the standard operating procedures (SOP) for NA isolation from old tissue blocks requires further advancement because of the inadequate quality of the obtained NAs^10^. Formalin can cause cross-linking of nucleic acids and proteins, which can affect PCR amplification. Furthermore, the quality of NAs extracted from FFPE samples can deteriorate over time, and this is influenced by the pH value of the fixative used for storage, although the tissue morphology is well preserved^11^. Thus, using FFPE samples for molecular analysis can encounter problems, although formalin fixation of tissue specimens for histopathological analysis has many advantages^8,9^. In particular, when using core needle specimens for molecular biological analysis, the quantity as well as quality of samples often pose a challenge due to the low sample volume. Therefore, our newly developed device might be a promising tool to overcome these issues because it can provide fresh frozen samples of sufficiently high quality and quantity to perform molecular biological analysis, such as gene panel testing.

In terms of RNA analysis, there are both qualitative and quantitative problems in RNA derived from FFPE samples and their utility in RNA expression analysis are known to be impaired because of RNA modifications by the FFPE procedure^12^. Many studies have reported that total RNA from a subset of FFPE tissues has good quality enough to perform a wide variety of RNA-based expression research^13–17^. At the same time, the fact that global gene expression information derived from FFPE specimens possesses poor quality and poor detection sensitivity in comparison to frozen specimens has also been accepted by many researchers. Further the adverse consequences are often observed with increasing frequency among aged archival FFPE specimens. We previously demonstrated that RNA extracted from divided frozen samples obtained using the new device exhibited remarkable quality and sufficient quantity for RNA examination, including real time PCR^7^. Thus, the newly developed device might be useful for providing high quality samples for both RNA analysis and histopathological evaluation. However, in the present study, RNA analysis was not performed. Therefore, further study for RNA analysis using the new device in this setting will be required.

Tumor heterogeneity is generally observed in many types of cancer where a variety of tumor cells exhibit distinct morphologic and phenotypic characteristics^18^. PCa was also believed to be a tumor characterized by a heterogeneous phenotype. Multifocal PCa were identified among 50% to 90% of patients who underwent radical prostatectomy which aligned with greater grade, stage, and rate of recurrence compared to unifocal PCa^19^. Ruijter et al. investigated the histological heterogeneity of multifocal diseases using a series of 61 whole mount radical prostatectomy specimens, and found that 72% of the specimens had multifocal disease and 84% had tumors representing multiple grades^20^. Thus, different tumor foci within the same PCa patient are thought to be pathologically distinct^21^. Meanwhile, recent literature illustrated that a major fraction of PCa exhibited signs of multiclonality, which means that genetically distinct and independently arising tumor clones do coexist^22^. PCa with metastasis also exhibits greater level of structural and molecular variety that aligns with higher resistance to systemic interventions. This increased intra-tumor heterogeneity (ITH) of PCa plays a significant role in diagnosis and offers significant challenges in the accomplishment of molecular research^23^. Thus, ITH in PCa is a highly significant phenomenon in comprehending tumor progression and also for finding out the clinical progression of disease and devising tailor-made intervention protocol for PCa. Thus it is very imperative to take into account the impact of ITH in arriving at the clinical decision when treating primary and as well as metastatic lesions, because a better knowledge about ITH will aid in the development of superior diagnostic tools and appropriate biomarkers and most importantly in selection of apt therapeutic strategy which will yield a better prognosis. In this context, our new device might provide a key to overcoming these challenges, since a fresh frozen sample of a divided tissue obtained using this device would be adequate for molecular biological analysis including gene panel testing.

There are several limitations in this study. First, it is important for users to acknowledge that there is a possibility that histopathological assessment could be affected by reducing the sample size since the mechanism of the new device is to divide one core needle biopsy specimen into two spatially matched samples. Second, the results of gene panel test and histopathological diagnosis when using the new device were not directly compared with standard methods where DNA was obtained from FFPE samples. Therefore, further study is required to ascertain the advantage of the new device compared to the current usual method.

In conclusion, our novel device for longitudinally dividing core needle prostate biopsy specimens provided paired mirror image-like tissue for gene panel and pathology testing. When obtaining prostate CNB specimens in patients with suspected PCa, the novel device for dividing CNB tissue allowed performance of histopathological diagnosis in one of the divided samples, while gene panel testing could be concomitantly performed on the paired sample. The device has the potential to be a promising tool for obtaining appropriate tissue for genetic and molecular biological analyses, apart from histopathological diagnosis, helping to advance personalized medicine.

## Materials and methods

### Patients and tissues

Forty consecutive patients with suspected PCa between November 2019 and July 2020 were included in this study. Patients were assessed with transrectal ultrasound-guided prostate CNB under local anesthesia. Macroscopically tentatively eligible specimens obtained by prostate CNB were divided into two pieces by the new device. One piece from the divided tissue was submitted for histopathological examination, and the other piece was used for molecular-biological analysis, including gene panel testing. The institutional review board (IRB) of Kyoto Prefectural University of Medicine (ERB-G-87) approved this research and was performed in accordance with Declaration of Helsinki. Written informed consent was obtained from every patient before study enrollment.

### Prostate biopsy

The multiparametric MRI (mpMRI) examinations including T2-weighted, dynamic contrast-enhanced, diffusion-weighted images and apparent diffusion coefficient maps were obtained by a 3 T MRI unit. The images were analyzed by trained radiologists using the Prostate Imaging-Reporting and Data System Version 2 (PI-RADS v2). MRI/US fusion biopsy was usually performed in patients with cancer focus (PI-RADS v2 score 3, 4 or 5) using computer-assisted MRUS-image fusion system with real-time 3D-transrectal ultrasound (TRUS) organ-tracking technology (Koelis, La Tronche, France). In MRI/US fusion biopsy, two biopsy cores were basically obtained from each target lesion and systematic biopsy (total 10 cores (5 cores/lobe)) was subsequently carried out. Systematic biopsy only was employed in patients without cancer focus on mpMRI. In patients who were highly suspected with advanced prostate cancer by several clinical findings such as an abnormally high PSA value (for example, 4504 ng/mL in this study) and mpMRI findings where cancer focus was totally occupied in prostate gland, the number of biopsy cores were reduced to minimize adverse effects by physicians’ decision according to the actual clinical setting.

### Procedure of the new device

The tissue extracted through prostate CNB (18G) was segregated into two portions using the new device as previously described^7^. Briefly, the procedure is performed as follows: (1) expose the biopsy notch (BN) placed distally in the needle, (2) position the tissue side of BN down on the device with a setting paper along the needle guide, (3) gently withdraw the needle leaving behind the tissue on the paper, (4) replace the device cover and press the lid with the sharp cutter downward, and (5) open the device and remove the divided tissues from the device. One piece of the divided sample was immediately stored in liquid nitrogen for subsequent gene panel testing and the other was fixed with formalin for histopathological diagnosis.

### DNA extraction and gene panel testing from divided tissues

Total DNA was separated through a DNeasy mini kit according to the manufacturer’s instruction (Qiagen Inc., Valencia, CA, USA). Extracted DNA was submitted for gene panel testing named Oncomine Comprehensive Assay v3 (Thermo Fisher Scientific Inc., MA, USA), and TruSight Oncology 500 (Illumina Inc., CA, USA). Quality assessment was performed according to each manufacturer’s protocol.

## Supplementary Information

Supplementary Information.

## Data Availability

The datasets used and/or analyzed in the current study are available from the corresponding author on reasonable request.
