# Supplementary material for: Novel device for dividing core needle biopsy specimens to provide paired mirror image-like tissues for genetic and pathological tests
Source: Sci Rep. 2023 Apr 24;13:6610. doi: 10.1038/s41598-023-33776-x (PMC10126131; doi:10.1038/s41598-023-33776-x)
Supplement: Supplementary file 1 — Supplementary Information. [file 41598_2023_33776_MOESM1_ESM.docx]

**Supplementary Material**

**Title:** Novel device for dividing core needle biopsy specimens to provide paired mirror image-like tissues for genetic and pathological tests

**Authors:** Yuichi Nakamura*, Keisuke Tsuji*, Takumi Shiraishi, Satoshi Sako, Ryota Ogura, Hideto Taga, Yuta Inoue, Munehiro Ohashi, Saya Ueda, Takeshi Yamada, Takashi Ueda, Atsuko Fujihara, Fumiya Hongo, Osamu Ukimura

* These two authors contributed equally to this work.

**Institution:** Department of Urology, Kyoto Prefectural University of Medicine, Kyoto, Japan

**Address:** Kawaramachi-Hirokoji, Kamigyo-ku, Kyoto 602-8566, Japan

TEL: +81-75-251-5595

FAX: +81-75-251-5598

**Correspondence:** Takumi Shiraishi, M.D., Ph.D.

Department of Urology, Graduate School of Medical Science, Kyoto Prefectural University of Medicine, Kawaramachi-Hirokoji, Kamigyo-ku, Kyoto 602-8566, Japan

TEL: +81-75-251-5595

FAX: +81-75-251-5598

E-mail: takumi14@koto.kpu-m.ac.jp

**Supplemental Figure**


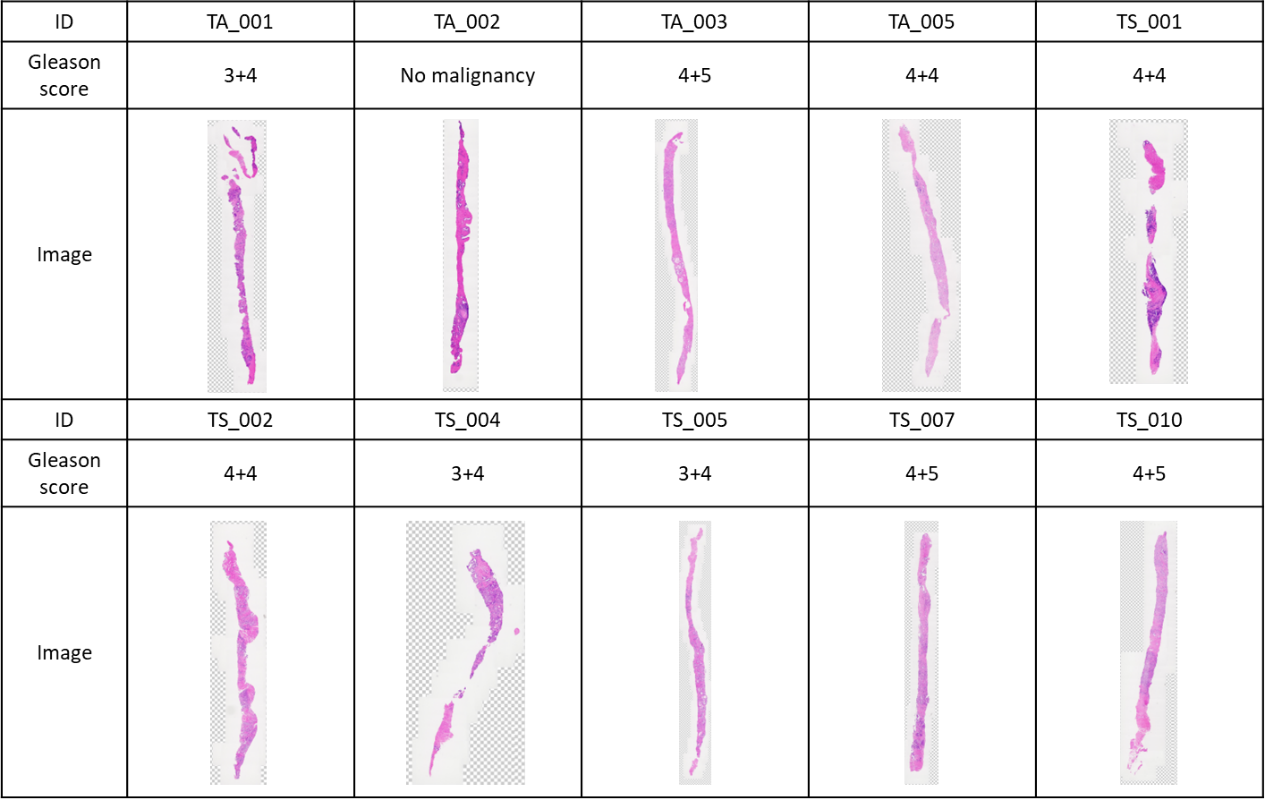


**Supplemental Figure 1. Representative images of divided samples stained by hematoxylin-eosin**

Representative images of divided samples stained by hematoxylin-eosin for histopathological diagnosis. The IDs in the figure correspond to the IDs in Table 2 and 3.
